# Optimising clinical trial management in Spain: The CARABELA-Clinical trials framework integrating healthcare models and subject experiences

**DOI:** 10.1016/j.conctc.2025.101544

**Published:** 2025-09-01

**Authors:** Carlos Almonacid, Borja G. Cosío, Xavier Muñoz Gall, Manuel Santiñà Vila, Jaime Signes-Costa, José Luis Velasco Garrido, Mercedes Luz, Marta Rodríguez, Alberto Godos, Ana Pérez Domínguez, Eunice Funenga, Carmen Moreno, CARABELA-CT Scientific Committee, Carlos Almonacid, Borja G. Cosío, Jaime Signes-Costa, José Luis Velasco Garrido, Juan Luis García Rivero, Manuel Santiñá Vila, Xavier Muñoz Gall, Alberto Godos, Alejandra Moreno, Ana Gómez, Ana Pérez Domínguez, Carmen Moreno Redondo, Carmen Vázquez, Eunice Funenga Fitas, Marta Rodríguez, Mercedes Luz, Raquel Rodríguez, Juan Luis García-Rivero

**Affiliations:** i Hospital Universitario Puerta de Hierro Majadahonda, Spain; j Hospital Universitario Son Espases, Spain; k Hospital Clínico Universitario de Valencia, Spain; l Hospital Universitario Virgen de La Victoria, Spain; m Hospital Universitario Marqués de Valdecilla, Spain; n Sociedad Española de Calidad Asistencial, Spain; o Hospital Universitario Vall D’Hebron, Spain; p Departamento Médico, AstraZeneca Farmacéutica, Spain; a Puerta de Hierro Majadahonda University Hospital, Calle Joaquín Rodrigo, 1, Majadahonda, 28222, Madrid, Spain; b Son Espases University Hospital-IdISBa and CIBERES, Carretera de Valldemossa, 79, Nord, 07120, Palma de Mallorca, Spain; c Vall D'Hebron University Hospital, Passeig de La Vall D'Hebron, 119, Horta-Guinardó, 08035, Barcelona, Spain; d Spanish Society for Healthcare Quality, Calle Uría 76, 1, Oficina 1, 33003, Oviedo, Spain; e Clínico University Hospital, INCLIVA, Avenida de Blasco Ibáñez, 17, El Pla Del Real, 46010, Valencia, Spain; f Virgen de La Victoria University Hospital, Campus de Teatinos, S/N, Puerto de La Torre, 29010, Málaga, Spain; g AstraZeneca Medical Department, Puerto de Somport 21-23. C.P: 28050, Madrid, España, Madrid, Spain; h Marqués de Valdecilla University Hospital, IDIVAL, Avenida de Valdecilla, S/n, 39008, Santander, Spain

**Keywords:** Clinical trial, Subject, Healthcare models, Healthcare quality indicators, CARABELA, Improvement areas, Solutions

## Abstract

Clinical trials (CT) are the framework upon which novel treatments' safety and efficacy are assessed. The CARABELA-CT initiative aims to optimise Spanish CT procedures by improving efficiency, quality, and subject well-being. To this, it characterised CT healthcare models, identified improvement areas, proposed solutions, and gathered important insights from CT subjects. CARABELA-CT took a three-phase approach. Phase I involved CT healthcare models’ characterisation, including all clinical investigation processes in Spain, across six pilot hospitals, identifying improvement areas and solutions. Phase II validated these findings, defining key healthcare quality indicators. Phase III focused on dissemination and implementation. Additionally, seven randomly invited CT subjects participated in a focus group to share their experiences regarding communication, coordination, and CT impact on quality of life. Three CT healthcare models were identified, distinguished according to access, infrastructure, and resources. Twelve improvement areas were defined, leading to 38 solutions addressing subject education, professional training, organisation, protocols, resources, and technology. Twenty-four healthcare quality indicators were established to monitor CT processes. Subject experiences highlighted the need for clearer communication, digitalised information, and improved subject support, and revealed the emotional and psychological benefits of CT participation, despite the challenges. CARABELA-CT provides a comprehensive framework to enhance Spanish CT processes. This initiative integrates potential solutions in improvement areas in CT development and the corresponding healthcare quality indicators, and prioritises subject experiences to foster efficiency, participant engagement, and a sustainable, patient-centric clinical research model. These findings contribute to the continuous improvement of CT management, ultimately optimising research execution and healthcare outcomes.

## Highlights

• Clinical trials (CT) are crucial for evaluating treatment efficacy and safety.
• CARABELA-CT aimed to optimise CT processes and enhance participant experience.
• Three CT care models were identified in Spanish hospitals.
• Twelve improvement areas and 38 solutions were prioritised for CT optimisation.
• A subject-centred approach highlighted key needs in communication and equity.

## 1 Introduction

Progress in modern medicine relies heavily on clinical trials (CT), as these studies provide a framework for evaluating the efficacy and safety of novel treatments [[1], [2], [3]] and play a pivotal role in enabling the development and validation of innovative therapeutic approaches in a wide range of diseases. They are conducted to assess safety and efficacy in phases, running from phase 0 (micro-dosing) to phase IV (post-marketing surveillance), passing through phase I (safety and dosage), phase II (efficacy and side effects), and phase III (confirmation and comparison). Participation in CTs is essential, not only because it accelerates the discovery of new treatments, but also because the insights gathered help to refine medical protocols, enhance patient care quality, and ensure better health outcomes [4,5]. Moreover, the findings derived from these trials significantly influence the design of future treatments, contributing to improved health standards and quality of life for individuals worldwide.

In recent years, the relevance of CTs has grown considerably due to rapid advancements in medical technologies and devices, innovative treatments, and the increasing demand for more personalised therapeutic solutions [1,6,7]. These developments underscore the vital role of subject participation in CTs. The individuals who take part in these trials become key contributors by providing invaluable data that directly impacts on the development of therapies and improves overall health outcomes. Furthermore, CT may be the only way to address certain conditions when approved treatments are not yet available, highlighting the essential role of subject involvement in these studies [[8], [9], [10]]. Ensuring an optimal experience for subjects is therefore of utmost importance, and a research process that prioritises their well-being and adheres to rigorous ethical and operational standards is essential.

Regulatory bodies such as the European Medicines Agency and the U.S. Food and Drug Administration play a critical role in safeguarding the integrity of CTs. These agencies oversee and approve trials to ensure compliance with international quality standards and enforce regulations that protect participants [2,11,12]. Similarly, trial sponsors—typically pharmaceutical companies or research organisations—are responsible for managing and supervising all aspects of the trial process, from selecting investigational sites to analysing results. They ensure that trials meet the highest standards of quality and ethical conduct and work towards advancing the development of innovative and effective therapeutic solutions [1,13].

As part of the range of CARABELA initiatives aimed at transforming the current management of different diseases and clinical situations [14], CARABELA-CT contributes to optimising CT procedures, ensuring quality at each stage and guaranteeing subject satisfaction and safety. It seeks to improve current healthcare models involving CT subjects and clinical research processes in Spain, all of which have been analysed from the perspective of both the subjects and the researchers. The CARABELA-CT initiative focused on the planning, implementation and development of phase II and III CTs with pharmaceutical products, aiming to enhance efficiency during their development, optimise procedures, and ensure compliance with the highest standards of clinical research, while consistently prioritising subjects’ well-being. The hospitals involved in this initiative, the Sociedad Española de Calidad Asistencial and AstraZeneca have produced this report on: i) the characteristics of clinical investigations, in order to define a standardised, efficient and professionally coordinated process; ii) the solutions suggesting improvement areas in this context, to ensure the comprehensive optimisation of clinical investigation and a better subject experience; iii) a healthcare quality indicator-based evaluation model aimed at following up the impact of these organisational and process solutions; and iv) the strategies needed to implement and disseminate these results, ultimately promoting a management model that will serve as a reference for the rest of the medical community responsible for developing CT.

## 2 Material and methods

CARABELA-CT initiative was designed as a three-phase process based on the general CARABELA methodology [14] during which the corresponding Scientific Committee, composed of researchers from six participating pilot centres and an expert in quality indicators, held several validation meetings (Fig. 1). Specifically, the aim of phase 1 (characterisation) was to design an appropriate clinical practice-based healthcare process within clinical investigation that met applicable Spanish and European regulatory frameworks. As part of this phase, the Scientific Committee defined an “ideal” process to characterise CT subjects and procedures based on Good Clinical Practice guidelines and relevant regulatory and ethical standards. This “ideal” model comprised five phases ranging from CT planning and feasibility study to start-up, development, monitoring, follow-up, and close-out (Fig. 2; Supplementary Material). Three key criteria (access to a CT unit [CTU]; infrastructure and resources; and CT type [phases I–IV]) were considered to identify the different CT care models across the six pilot centres. A process mapping exercise was then carried out at each site to assess their existing workflows in relation to the “ideal” model. These teams conducted structured workshops involving clinicians and investigators from each centre to identify improvement areas and potential solutions. In the following phase (phase 2 - validation), the Scientific Committee reviewed the findings from each centre, validated and prioritised the identified improvement areas and proposed solutions, and defined a set of corresponding healthcare quality indicators. Finally, phase 3 (dissemination and implementation) resulted in the generation of a Playbook in which all the initiative data were included as a guide for CT development across Spain. The CARABELA-CT Playbook is a digital feasibility platform that facilitates regional workshops in which healthcare models, improvement areas and potential solutions are specifically refined.

**Fig. 1:**
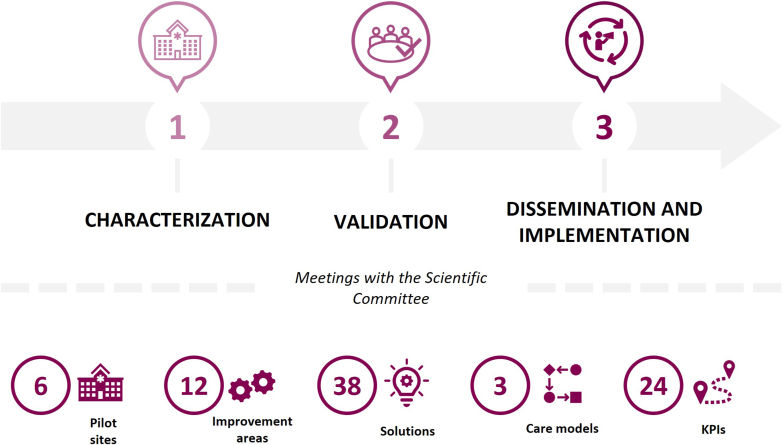
Summary of the CARABELA-CT initiative methodology and results.

**Fig. 2:**
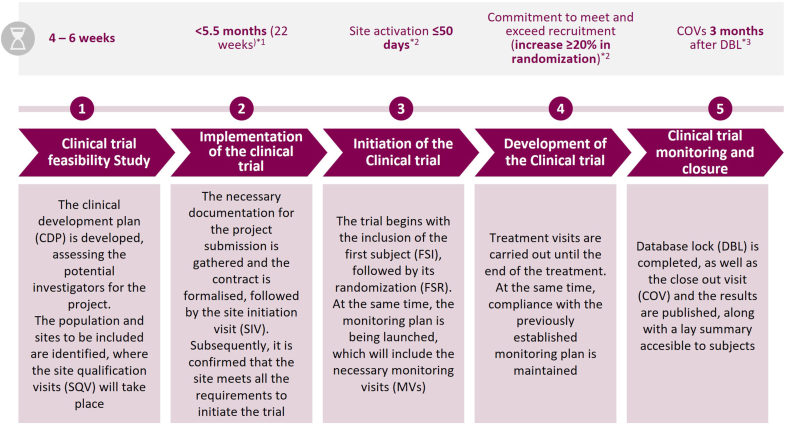
The “ideal” process for the development of a CT. ∗^1^Based on EU Clinical Trials Register experience throughout 2024 [2,12,16]. CT: clinical trial. [1] Regulatory harmonisation of clinical trials in the EU: Clinical Trials Regulation to enter into application and new Clinical Trials Information System to be launched. Available at: https://www.ema.europa.eu/en/news/regulatory-harmonisation-clinical-trials-eu-clinical-trials-regulation-enter-application-and-new-clinical-trials-information-system-be-launched. [2] European Medicines Agency. Clinical Trials in the European Union. Available in: Clinical Trials in the European Union – EMA https://euclinicaltrials.eu/. [3] ICH HARMONISED GUIDELINE GUIDELINE FOR GOOD CLINICAL PRACTICE E6(R3). INTERNATIONAL COUNCIL FOR HARMONISATION OF TECHNICAL REQUIREMENTS FOR PHARMACEUTICALS FOR HUMAN USE. Final version Adopted on January 06, 2025. Available at: https://database.ich.org/sites/default/files/ICH_E6%28R3%29_Step4_FinalGuideline_2025_0106.pdf.

In parallel with these activities, focus centred on the “subject's voice” in a focus group in which seven randomly invited subjects participating in CT from different regions in Spain shared their experiences in the context of clinical investigation. A series of qualitative and quantitative questions were discussed, encompassing two key categories: i) communication and coordination; and ii) feelings and quality of life. The former addressed the interaction between healthcare professionals and subjects throughout a CT and the latter concerned the day-by-day routine of a CT subject, including aspects related to work, social, and family areas, and aimed to provide a more detailed picture of how these areas and thus the subject's general well-being and quality of life may be influenced during a CT. Finally, the opinions gathered through a questionnaire were used to construct an “empathy map” of the subjects' feelings throughout all the CT phases.

## 3 Results

### 3.1 Characterisation of the CT healthcare models in Spanish hospitals

Three different models currently existing were identified (Table 1). In model A, applicable to CT phases I, II, III, and IV, both a CTU (either department-specific or centre-shared) and a proper infrastructure offering the human and material resources needed to develop a CT are available. This model has at least one study coordinator who can assume data manager functions and a study nurse. Model B resembles the first but lacks access to a CTU, and only applies to CT phases II, III and IV. Finally, in model C, applicable to CT phases III and IV, access to a CTU is a possibility, but the infrastructure and human and material resources available to develop the CT are not entirely sufficient.

**Table 1:** CT development models identified in CARABELA-CT.

	Model A	Model B	Model C
Access to CT unit	Available	Not available	Potentially available
Infrastructure and human and material resources	Full access^a^	Full access^a^	Partial lack of access^b^
CT phases	I–IV	II, III, and IV	III and IV

CT: clinical trial.

a Includes a study coordinator who can assume data manager functions and a study nurse.

b Solutions, resources, and training must be provided to meet CT requirements.

### 3.2 Improvement areas and potential solutions

Twelve improvement areas influencing these CT models were defined. In correspondence, 38 potential solutions emerged to contribute to the continuous improvement and optimisation of each CT model (Table 2). These solutions were classified into six categories: i) subject education; ii) professional training; iii) organisation; iv) protocols; v) resources; and vi) technology. The improvement areas were prioritised according to their impact on daily clinical practice and the practicality of dealing with them. The solutions were also prioritised on the basis of their impact and how quickly they could be implemented.

**Table 2:** Improvement areas defined and potential solutions proposed within the CARABELA-CT initiative.

Improvement area	Potential solution	Solution category
1. Lack of participation of PC in identifying CT subjects	1. Incorporate the PC physician as a co-investigator (e.g.: subject referral) listed as member of the site staff	Organisation
	2. Establish virtual clinical trial consultations with PC to make them aware of the existence of the trials and the inclusion/exclusion criteria	Organisation
	3. Create a referral network in PC, designating reference professionals to facilitate coordination with the site staff in the identification and referral of subjects for CT	Organisation
2. Lack of communication with PA for the identification of CT subjects	4. Establish a communication channel with the PA for the identification of subjects	Organisation
	5. Publish on the websites of the PA the ongoing CT in the centres	Organisation
3. Need to optimise communication and coordination between the site and the sponsor	6. Establish a communication plan between the site and the sponsor to inform subjects on the treatment administered during the CT and ensure they are recorded in the MR	Protocols
	7. Streamline the query resolution circuit for consulting the sponsor on clarifications on CT inclusion/exclusion criteria	Protocols
	8. Create a shared repository between the site and the sponsor with specific information/documentation on the trials and feasibilities (e.g. incidence, subject characteristics, etc.) to avoid duplication in requests for feasibility information and common documents in different trials	Technology
	9. Classify the risk of queries, assigning them a longer or shorter resolution time depending on their relevance	Organisation
**4. Lack of visibility in CTs**	10. Encourage the use of a department-specific annual clinical research report to verify feasibility information	Protocols
	11. Disseminate CT information on social media/media and CT registry websites (e.g., REec)	Organisation
**5. Difficulty in identifying and selecting subjects for CT**	12. Establish a decision tree that assists the site staff in prioritising the participation of CT subjects	Organisation
	13. Create a tool that includes inclusion/exclusion criteria to facilitate the identification of subjects	Technology
	14. Integrate treatment received from previous CTs into the site's database algorithms to identify eligible subjects and expedite their inclusion in CT	Technology
	15. Develop a schedule based on recruitment expectations that takes into account seasonal variations and holiday periods, as well as patterns of exacerbation and vaccine use, especially in experimental biological treatments where these are limiting factors	Organisation
	16. Promote collaboration between the site staff and the Pharmacy to obtain a list of subjects who have completed a certain treatment (e.g., biologic), optimising the evaluation of their eligibility in other CTs	Organisation
**6. Difficulty in developing CT procedures**	17. Encourage the PI to review the protocol design to bring it more in line with the usual practice/real subject	Organisation
	18. Align the procedures in the selection visit/initiation visit according to the protocol so that they coincide with the daily care activities	Organisation
	19. Create digital templates/checklists by the sponsor detailing procedures, order, and data to be collected in each visit.	Technology
	20. Reinforce the figure of the study coordinator/data manager to ensure continuous and timely data entry	Resources
	21. Create a contract/framework clause with the site that streamlines management and signature procedures	Organisation
	22. Conduct clinical trial activities in the afternoon, whenever possible, to optimise the time of both the site staff and the subjects, as well as the spaces available in the site	Resources
	23. Report the participation of subjects in clinical trials in the MR	Technology
**7. Overload of site staff**	24. Define the contents and times of face-to-face and remote monitoring visits to facilitate site staff organisation	Organisation
	25. Assign a specific day for CT and allow the site staff to focus exclusively on these tasks, without interfering with their care responsibilities	Organisation
	26. Rationalise and simplify the number of training courses to speed up the site staff learning process (e.g. in the handling of specific devices/systems for each test)	Professional training
**8. Need to optimise facilities, human resources and equipment**	27. Prepare a document during the feasibility and/or site selection visit identifying the human and material resources required for the trial	Resources
	28. Coordinate the shipment of materials with the sponsor to ensure an expiration date (especially laboratory kits) close to the activation of the site, minimising waste due to expiration	Protocols
	29. Establish a shared trial schedule that ensures the availability of material for each trial and the allocation/reservation of spaces for subject visits, to maximise site space	Organisation
	30. Identify medical procedures that create a bottleneck in the facility	Resources
	31. Enable a CT area that is easily accessible to subjects	Resources
**9. Inefficiency in handling and management by vendors**	32. Establish a circuit to validate the status and configuration of devices and to confirm their correct receipt and return	Protocols
	33. Provide 24-h phone/email support for the resolution of problems and doubts, in local language if possible	Resources
**10. Limitations in information systems in the coding of clinical trial subject data**	34. Implement a tool that properly codes and identifies subjects who meet the trial inclusion/exclusion criteria, facilitating their early identification (pre-screening)	Technology
	35. Include an IT figure to assist in the coding process for subject identification in the site databases	Technology
**11. Failure to deliver the lay summary to clinical trial subjects**	36. Highlight at the closing visit the existence of the lay summary, also mentioned in the information and informed consent form that has been given to the subject at the beginning of the trial, and ensure that subjects receive the clinical trial information and understand their participation in the clinical trial	Organisation
	37. Follow-up by the sponsor together with the site staff of the final communication of the results (lay summary) to the subjects	Protocols
**12. Failure to report subject experience in the MR**	38. Conduct, analyse and exploit questionnaires on PREMs and health outcomes that can be integrated into the subject's MR, adapted to the profile of the subject in each pathology.	Resources

CT: clinical trial; IT: information technology; MR: medical record; PA: patient associations; PC: primary care; PREMs: Patient-Reported Experience Measures; REEC: Registro Español de Ensayos Clínicos.

### 3.3 Identification of healthcare quality indicators

A total of 24 healthcare quality indicators were used to assess the different CT models across the different centres. These indicators were divided into three main categories: i) structural indicators on material and human resources and available infrastructure; ii) healthcare process indicators that measure how adequate all the processes established are; and iii) result indicators assessing the effectiveness (healthcare outcomes), efficiency, and quality of care (subject security, continuity of care, equity.). All the indicators finally defined are listed in Table 3.

**Table 3:** List of healthcare quality indicators validated in the CARABELA-CT initiative.

STRUCTURAL INDICATORS	
Indicator	Description
Primary Care (PC) investigators	Existence of clinical investigators from each Health Area in PC who refer subjects for CT (Yes/No)
Department-based investigators	Existence of clinical investigators from each Health Area in each department for the identification of CT subjects (Yes/No)
Investigators from other centres	Existence of at least one clinical investigator from each Health Area in the other sites for the identification of CT subjects (Yes/No)
Collaboration with Patient Associations (PA)	Existence of a collaboration agreement with PA for the participation of CT subjects (Yes/No)
Notifications in electronic medical records (MR)	Existence of a notification in the electronic MR of CT subjects (Yes/No)
Clinical Trials unit (CTU)	Existence of a CTU at the site (Yes/No)
Quality certification	Existence of quality certification in the CTU (e.g., ISO) (Yes/No)
Time allocation for clinical research	Existence of time assigned for research in the agenda of the specific physician (Yes/No)
Study coordinator and study nurse	Existence of at least one study coordinator and one study nurse in the centre/department (Yes/No)
Phase I unit	Existence of a certified phase I unit in the centre/department (Yes/No)
Physical space for monitoring	Existence of a space for monitoring visits (Yes/No)

PROCESS INDICATORS	

Time for contract finalisation	The management/completion of the contract prior to signatures for the conduct of CT initiation does not exceed 90 days (Yes/No)
Time for centre activation	The average time from the start visit to the activation of the site does not exceed 15 days (Yes/No)
Ready-to-enrol time	The average time from ready-to-enrol to the first subject included in the trial does not exceed 15 days (Yes/No)
Time for query resolution	Resolution of queries in ≤5 calendar days (Yes/No)
Time for DCF data entry	DCF data entry in ≤5 calendar days (Yes/No)
Principal Investigator (PI) presence in monitoring	Presence of the PI at monitoring visits (Yes/No)

RESULTS INDICATORS	

Subject dropout rate	Percentage (%) of subjects who leave the CT with subsequent follow-up (subjects leaving the CT with subsequent follow-up/total of subjects recruited)
Screening failure rate	Percentage (%) of screening failures (subjects with a screening failure/total of subjects recruited)
Subject satisfaction surveys (PREMs)	Conducting satisfaction surveys/PREMs questionnaires that evaluate the experience of subjects (Yes/No)
Recruitment commitment rate	Percentage (%) of subjects recruited vs. investigator recruitment commitment (total subjects recruited/total commitment)
Serious adverse event reporting	All serious adverse events are reported in less than 24 h (Yes/No)
Audit major findings	Number of major observations identified in audits in the last two years (*n*)
Protocol deviations	Number of protocol deviations (*n*)

CT: clinical trial; CTU: CT unit; DCF: data collection form; MR: medical record; PA: patient associations; PC: primary care; PI: principal investigator; PREMs: Patient-Reported Experience Measures.

### 3.4 The subject's voice: real-world experiences

**Fig. 3:**
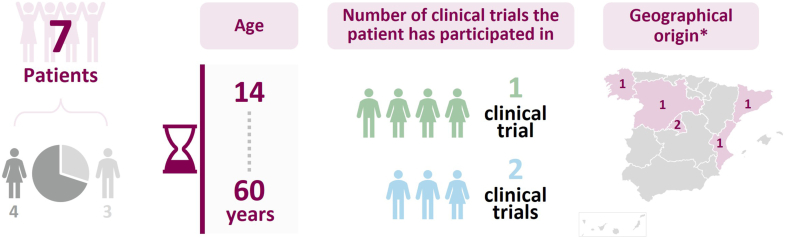
Characteristics of participants in the *Subject's Voice* focus group. ∗No data available on the geographical origin of one of the patients. CT: clinical trial.

The characteristics of the subjects participating in the focus group are shown in Fig. 3.

The need for a clear, detailed, comprehensible and continuous communication of CT-related aspects with healthcare professionals was identified as a main improvement area. Subjects were informed about the existence of a CT in diverse ways. Most were informed at the consultation, but some others proactively proposed their participation after being informed otherwise. Most participants stated that the information received was transparent, although one would have preferred to receive detailed information about subject selection criteria and CT design, in order to ensure equity and diversity, and information about the CT design.

Subjects found that the materials provided were complex and stated a preference for digital, interactive formats over extensive physical documents. This lack of digital support was also noted in follow-up visits. Furthermore, although the reported dedication and professionalism of various healthcare professionals in query resolution was appreciated, the designation of a single case manager would have ensured a quick, personalised response.

Finally, the subjects reported initial stress and uncertainty due to limited information about their treatment. They emphasised that they constantly felt the need for updates, particularly regarding preliminary results and potential side effects. The scarcity and lack of clear information at key moments generated uncertainty and, in some cases, doubts about continuing participation in the trial.

Another improvement area related to exploratory testing. The participants felt that they should have received information on individual and CT global outcomes, in order to understand their impact on the CT. They emphasised the need for faster access to results and detailed feedback from professionals to enhance transparency and comprehension. They also reported delays and long waiting times in the monitoring appointments, especially when these involved more than one specialist and/or hospital area. This situation sometimes made it difficult to reconcile follow-up visits with the subject's daily routine, especially during working hours. Despite these areas that needed improvement, subjects emphasised the emotional and psychological benefits of their participation. Most agreed that the experience provided them with a sense of purpose and positively impacted on their well-being, especially those who noticed health improvements due to the trial follow-ups. Many expressed deep personal satisfaction, viewing the CT not only as a source of hope but also as their only treatment option. This made them feel grateful and optimistic, knowing they were receiving advanced care while contributing to future medical progress. Their involvement gave them a sense of purpose, empowerment, and personal satisfaction, especially those who noticed health improvements. They valued contributing to future medical progress while receiving advanced care.

**Fig. 4:**
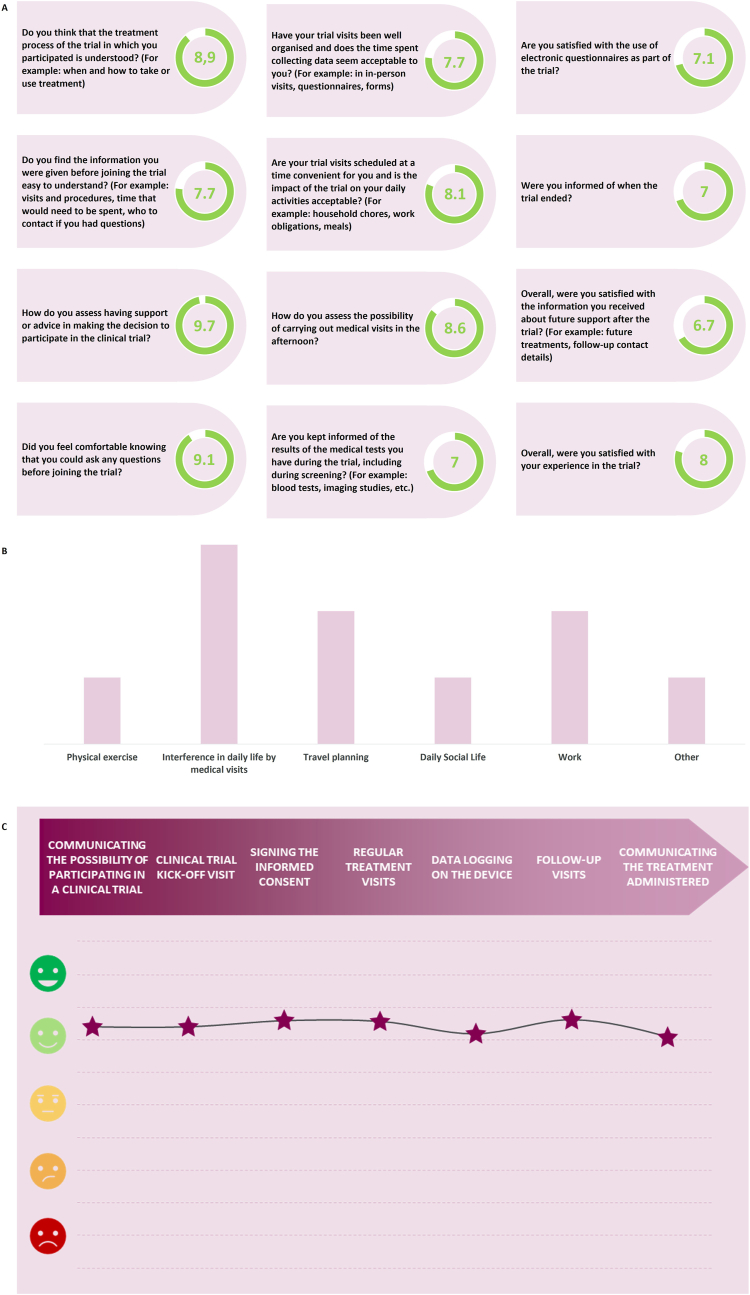
Quantitative results of the collection of the *Subject's Voice* data within CARABELA-CT. **A.** Subject satisfaction survey on clinical trial experience. **B.** Subject life areas affected as a result of participating in a CT. **C.** Empathy map showing the subject's emotional journey throughout the possible phases of a CT. This map shows the average of the feelings shared by the participants. CT: clinical trial.

The subjects also answered a series of quantitative questions regarding the information provided to them and the way they received it, queries resolution, monitoring visits, exploratory testing and questionnaires, and global satisfaction. The results are summarised in Fig. 4, which also includes their opinion about the impact of CT on different areas, and an empathy map. The latter shows how subjects generally maintained a stable mood, supported by informed consent and communication at the beginning of the trial, though repetitive data collection negatively affected them. Detailed feedback during follow-ups improved their mood slightly, while unclear treatment information caused occasional uncertainty.

## 4 Discussion

CTs are a crucial driver of medical progress. They provide the solid foundation needed to develop safe and effective solutions to evolving health challenges and are indispensable for generating high-quality clinical evidence [1,15]. In recent years, CT have become more refined and quality controls have improved significantly [2]. Moreover, the update of the ICH harmonised guideline for Good Clinical Practice ICH E6(R3), which will come into force in June 2025, incorporates novel recommendations that focus on enhancing trial quality, adopting a patient-centric perspective by involving key stakeholders, promoting flexible and adaptive trial designs for broader participant inclusion, and reinforcing ethical considerations to safeguard participants’ rights and well-being in line with evolving regulatory standards [16]. Consequently, it has become imperative to assess the implementation of quality-of-care programmes and gain a comprehensive understanding of the current processes of CTs. In this context, the CARABELA-CT initiative has concentrated its efforts on identifying models and providing a comprehensive understanding of the healthcare processes for subjects participating in CTs in Spanish hospitals.

The comprehensive characterisation process of the CARABELA-CT initiative, conducted across six pilot centres and with the participation of Sociedad Española de Calidad Asistencial, provided valuable insights into diverse scenarios by identifying three distinct models of care. These models differ, but the primary distinguishing factor is accessibility to a specific CTU, characterised by availability of infrastructure, the presence of a study coordinator who can assume data manager functions and a specialised nurse. Additionally, a broad set of healthcare quality indicators were collected and validated to help optimise the CT process. These indicators were tailored to each of the identified care models and processes, and solutions were implemented in the different categories. Addressing these improvement areas through the proposed solutions is essential to overcome existing challenges in CT management and enhance subject identification, selection, and recruitment. A comprehensive approach that integrates these elements improves overall efficiency and CT quality, while also addressing structural, clinical, and attitudinal barriers to trial participation, as described in previous research [8,10,17].

In fact, significant progress has been made in recent years with the introduction of guidelines and clinical research regulations [16] emphasising the importance of designing patient-centric CT that address subjects' needs, preferences, and experiences [9,10,17,18]. In this context, the CARABELA-CT initiative incorporated the real-life experiences of subjects in the “*subject's voice”* focus group, offering valuable insights into improvement areas that directly impact subjects' lives in Spain. Key improvement areas for including clearer communication and more equitable participant selection align with those identified in other studies [[18], [19], [20]]. Some subjects also emphasised the need for clearer and more accessible communication from professionals, particularly at critical moments, to reduce uncertainty and build trust. This feedback underscores the importance of ensuring that trial information is understandable, especially for groups like minors. It highlights the need to improve selection criteria in order to promote equity and diversity with the aim of engaging and recruiting underrepresented groups [19,20]. Many subjects also expressed a desire for greater transparency and open communication, as this fosters a stronger connection with researchers, a factor previously described as favourable in encouraging the recruitment and retention of subjects in trials [21]. Furthermore, engaging subjects through participatory activities, such as expert workshops for participants, was identified as a key motivational factor to enhance commitment and emotional well-being. Despite the challenges, trials represented for many not only hope but also the sole treatment option for their conditions, fostering gratitude, optimism, and empowerment gained through their involvement, while emphasising the human and scientific value of clinical trials.

A potential limitation of the study is that it primarily included subjects for whom the CT represented the only available treatment option, a factor which may introduce bias by excluding chronic subjects with more therapeutic alternatives who participate in trials out of commitment to science. However, it may also provide valuable insights for chronic participants who have exhausted all available treatment options. Another potential limitation was the small number of participants in the subject's voice initiative; however, its aim was exploratory and qualitative, intended to gather preliminary insight into subject's experiences in the context of CT.

CARABELA-CT has taken an innovative approach tailored to the diverse realities and existing CT care models across Spain. The strengths of this initiative include the involvement of a significant number of healthcare professionals with expertise in CT development and the inclusion of subjects’ perspectives and experiences. It addresses all levels of healthcare to overcome current barriers in CT design and execution, as well as the inclusion of subjects. Finally, in a subsequent practical phase, this initiative will be disseminated and implemented in the form of local workshops and continuous training programmes held in healthcare centres throughout Spain, to enhance the knowledge and attitudes of healthcare professionals involved in CTs and, more broadly, the improvement of CT processes across Spain through a comprehensive and integrated approach.

## CRediT authorship contribution statement

**Carlos Almonacid:** Writing – review & editing, Validation, Supervision, Investigation, Conceptualization. **Borja G. Cosío:** Writing – review & editing, Validation, Supervision, Investigation, Conceptualization. **Xavier Muñoz Gall:** Writing – review & editing, Validation, Supervision, Investigation, Conceptualization. **Manuel Santiñà Vila:** Writing – review & editing, Validation, Supervision, Investigation, Conceptualization. **Jaime Signes-Costa:** Writing – review & editing, Validation, Supervision, Investigation, Conceptualization. **José Luis Velasco Garrido:** Writing – review & editing, Validation, Supervision, Investigation, Conceptualization. **Mercedes Luz:** Writing – review & editing, Validation, Supervision, Investigation, Conceptualization. **Marta Rodríguez:** Writing – review & editing, Validation, Supervision, Investigation, Conceptualization. **Alberto Godos:** Writing – review & editing, Validation, Supervision, Investigation, Conceptualization. **Eunice Funenga:** Writing – review & editing, Validation, Supervision, Investigation, Conceptualization. **Carmen Moreno:** Writing – review & editing, Validation, Supervision, Investigation, Conceptualization. **Juan Luis García-Rivero:** Writing – review & editing, Validation, Supervision, Investigation, Conceptualization.

## Declaration of generative AI and AI-assisted technologies in the writing process

During the preparation of this work the authors used ChatGPT (OpenAI) in order to improve the readability and language of the manuscript. After using this tool/service, the authors reviewed and edited the content as needed and take full responsibility for the content of the published article.

## Funding sources

This work was supported by 10.13039/100019717AstraZeneca Farmacéutica Spain.

## Declaration of competing interest

The authors declare the following financial interests/personal relationships which may be considered as potential competing interests:

**All authors** declare support for medical writing assistance on this manuscript funded by 10.13039/100019717AstraZeneca Farmacéutica Spain. **All authors** also recognize the role that 10.13039/100019717AstraZeneca Farmacéutica Spain played as a co-organizer of the CARABELA-CT initiative, together with the hospitals and Scientific Societies involved in it. **Carlos Almonacid** has received grants from 10.13039/100004325AstraZeneca and 10.13039/100019720GSK and personal fees and non-financial support from 10.13039/100004325AstraZeneca, 10.13039/100019720GSK, TEVA, 10.13039/100019719Chiesi, 10.13039/100004339Sanofi, 10.13039/100030732MSD, and 10.13039/100016525FAES. **Borja G. Cosío** has participated in advisory boards, and has received grants and travel support from AstraZeneca, Boehringer Ingelheim, Chiesi, GSK, Menarini, Novartis, ROVI, Sanofi, and TEVA. **Xavier Muñoz Gall** has received fees as a speaker, scientific advisor or participant of clinical studies from AstraZeneca, Boehringer Ingelheim, Chiesi, FAES, Gebro, GSK, Menarini, Mundifarma, Novartis, Sanofi, and TEVA. **Manuel Sanitñá Vila** declares no conflict of interests. **Jaime Signes-Costa** has served as a consultant and received speakers' fees at advisory boards from Aflofarm, AstraZeneca, Boehringer Ingelheim; he has received institutional funding for trials and research from Boehringer Ingelheim and GSK; and he has received support for attending meetings and/or travel from Aflofarm, Boehringer Ingelheim, FAES, Menarini, and TEVA. **José Luis Velasco Garrido** has received personal fees from AstraZeneca, Sanofi-Genzyme and GSK; and non-financial support from AstraZeneca. **Mercedes Luz, Marta Rodríguez, Alberto Godos, Ana Pérez Domínguez, Eunice Funenga and Carmen Moreno** are employees at Departamento Médico, AstraZeneca Farmacéutica Spain. **Juan Luis García Rivero** has received speakers’ fees from GSK, AstraZeneca, Chiesi, Grifols and Sanofi; and consultancy fees from GSK, AstraZeneca, Grifols, ALK, and Sanofi.

## Figures and Tables

**figs1:**
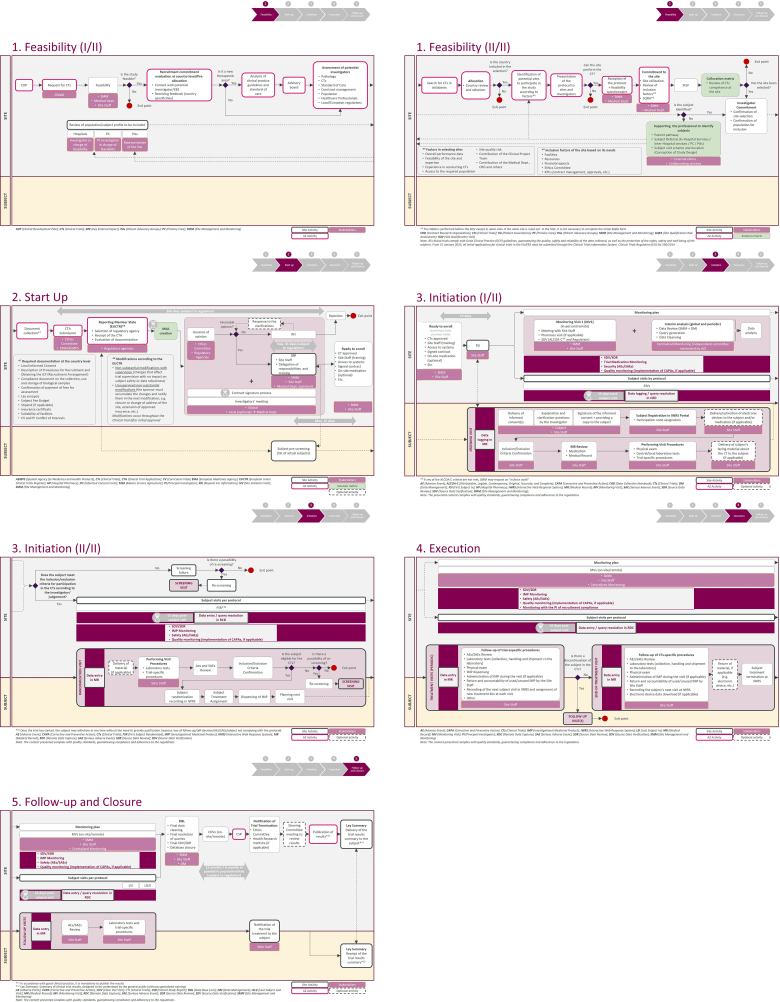


## Data Availability

All the relevant data are included within the manuscript

## References

[1] Kandi V., Vadakedath S. (2023). Clinical trials and clinical research: a comprehensive review. Cureus.

[2] Regulatory harmonisation of clinical trials in the EU: Clinical trials regulation to enter into application and new clinical trials information system to be launched. https://www.ema.europa.eu/en/news/regulatory-harmonisation-clinical-trials-eu-clinical-trials-regulation-enter-application-and-new-clinical-trials-information-system-be-launched.

[3] The Need for Awareness of Clinical Research NIH clinical research trials and you. https://www.nih.gov/health-information/nih-clinical-research-trials-you/need-awareness-clinical-research.

[4] Zvonareva O. (2023). Patient engagement in drug development: configuring a new resource for generating innovation. Crit. Public Health.

[5] Bombard Y., Baker G.R., Orlando E., Fancott C., Bhatia P., Casalino S., Onate K., Denis J.-L., Pomey M.-P. (2018). Engaging patients to improve quality of care: a systematic review. Implement. Sci..

[6] Biankin A.V., Piantadosi S., Hollingsworth S.J. (2015). Patient-centric trials for therapeutic development in precision oncology. Nature.

[7] Piantadosi S. (2024).

[8] Unger J.M., Cook E., Tai E., Bleyer A. (2016). The role of clinical trial participation in cancer research: barriers, evidence, and strategies. Am Soc Clin Oncol Educ Book.

[9] Arumugam A., Phillips L.R., Moore A., Kumaran S.D., Sampath K.K., Migliorini F., Maffulli N., Ranganadhababu B.N., Hegazy F., Botto-van Bemden A. (2023). Patient and public involvement in research: a review of practical resources for young investigators. BMC Rheumatol.

[10] Sacristán J.A., Aguarón A., Avendaño-Solá C., Garrido P., Carrión J., Gutiérrez A., Kroes R., Flores A. (2016). Patient involvement in clinical research: why, when, and how. Patient Prefer. Adherence.

[11] Liu C., Walker A.J., Translational Radiation Oncology (2023). Handbook for Designing and Conducting Clinical and Translational Research.

[12] European Medicines Agency (2001). Clinical trials in the european union. Available in: Clinical Trials in the European Union – EMA.

[13] Boissel J.-P. (2004). Planning of clinical trials. J. Intern. Med..

[14] Escalada J., Carretero Gomez J., Anguita M., de Sequera P., Garcia-Rio F., Davila I., Soto Bonel J.F., Garcia J.P., Rodriguez Ledo P., Barrena E., Fitas E., Prado A., Regadera L., Perez A., Mediavilla I. (2024). Enhancing the management of chronic diseases in clinical practice: the CARABELA methodology. J Healthc Qual Res.

[15] What are clinical trials and studies? Clinical trials and studies. https://www.nia.nih.gov/health/clinical-trials-and-studies/what-are-clinical-trials-and-studies.

[16] (2025). Ich harmonised guideline guideline for good clinical practice e6(r3). International council for harmonisation of technical requirements for pharmaceuticals for human use.

[17] Farah E., Kenney M., Kica A., Haddad P., Stewart D.J., Bradford J.-P. (2023). Beyond participation: evaluating the role of patients in designing oncology clinical trials. Curr. Oncol..

[18] Benvenuti S., Wang C.M., Borroni S. (2021). Perspectives, expectations, and concerns of European patient advocates on advanced therapy medicinal products. Front. Med..

[19] Carter-Edwards L., Hidalgo B., Lewis-Hall F., Nguyen T., Rutter J. (2023). Diversity, equity, inclusion, and access are necessary for clinical trial site readiness. J Clin Transl Sci.

[20] Kelsey M.D., Patrick-Lake B., Abdulai R., Broedl U.C., Brown A., Cohn E., Curtis L.H., Komelasky C., Mbagwu M., Mensah G.A., Mentz R.J., Nyaku A., Omokaro S.O., Sewards J., Whitlock K., Zhang X., Bloomfield G.S. (2022). Inclusion and diversity in clinical trials: actionable steps to drive lasting change. Contemp. Clin. Trials.

[21] Anastasi J.K., Capili B., Norton M., McMahon D.J., Marder K. (2023). Recruitment and retention of clinical trial participants: understanding motivations of patients with chronic pain and other populations. Front Pain Res (Lausanne).

